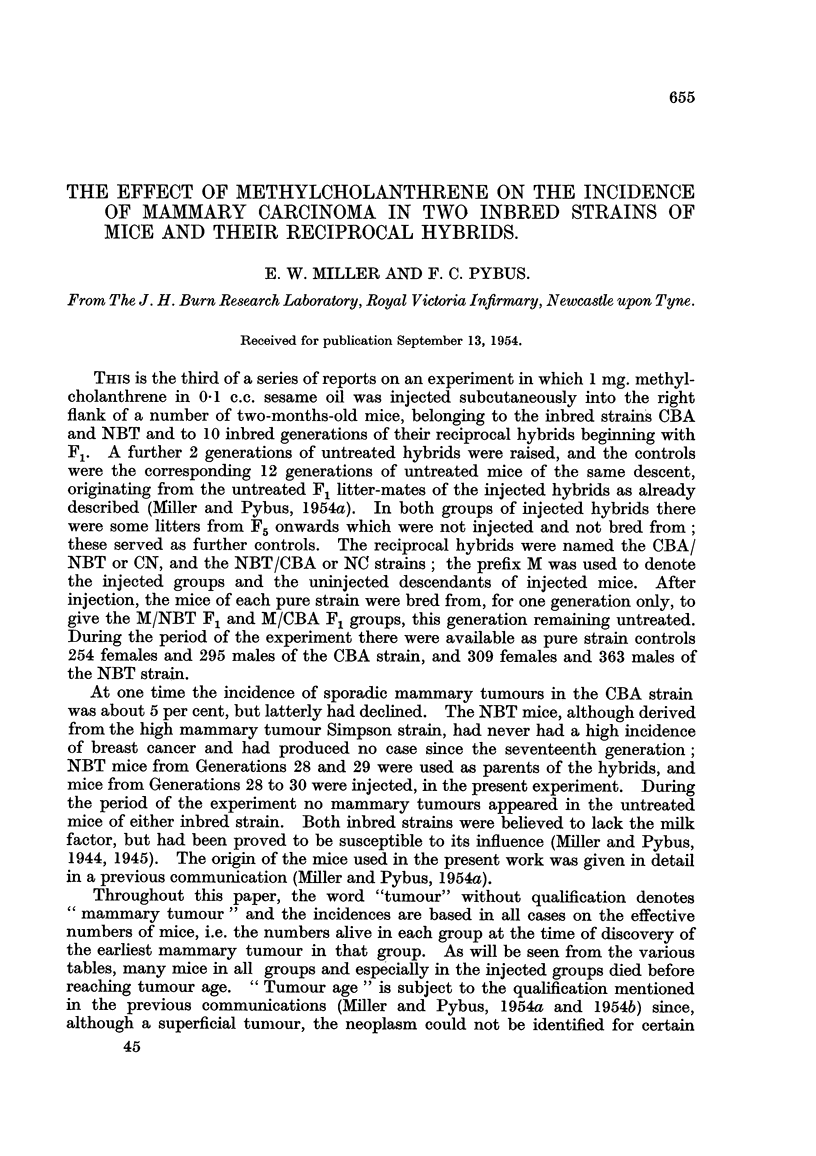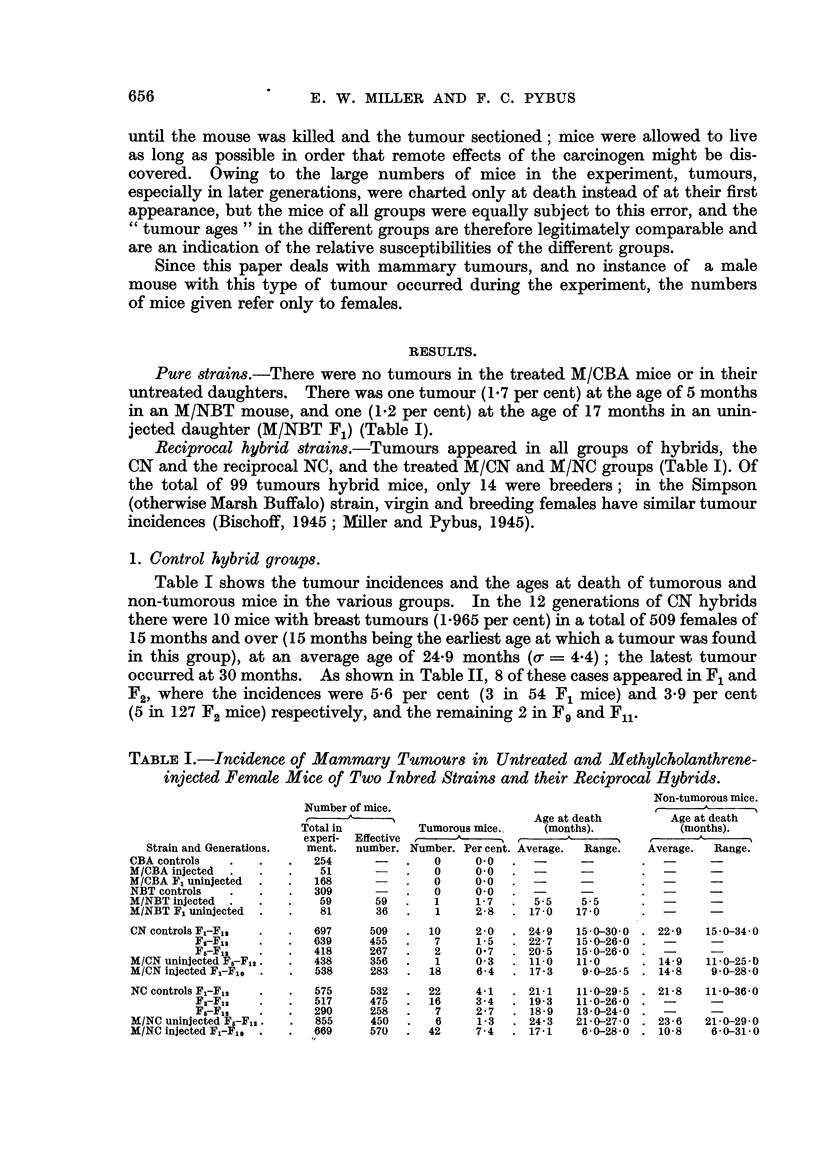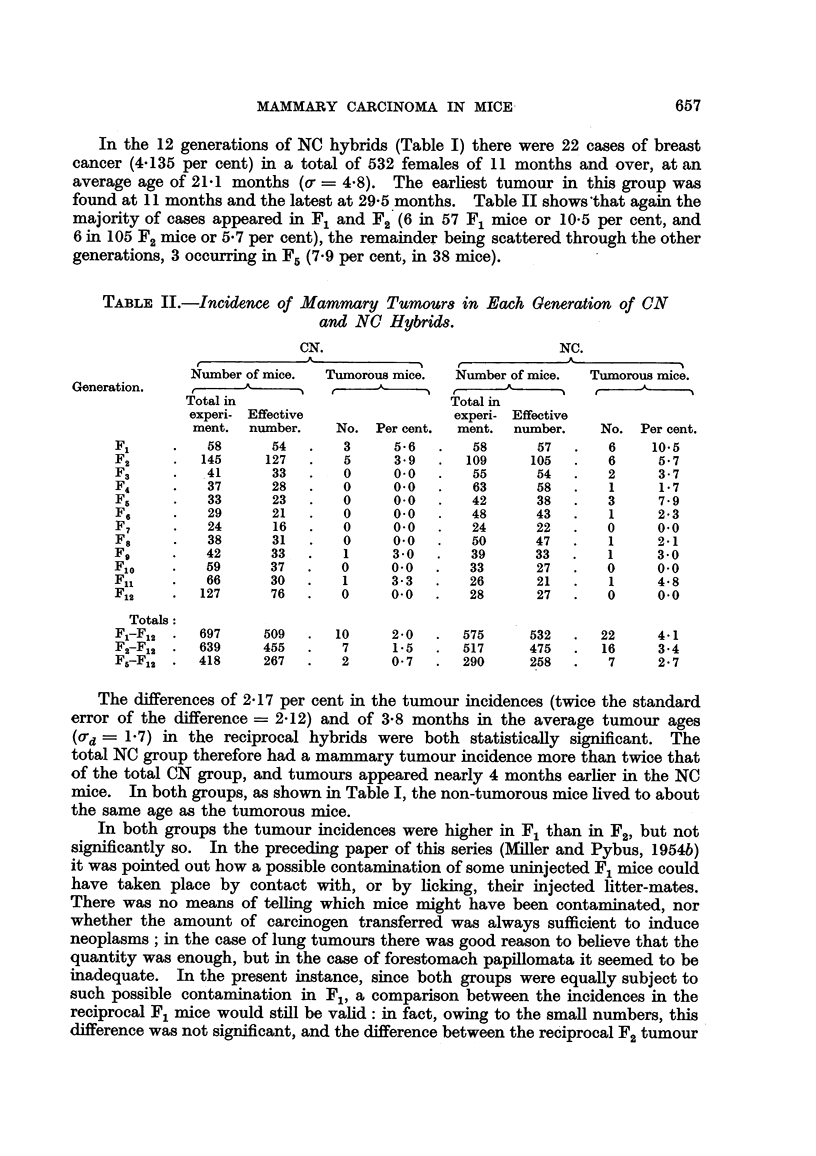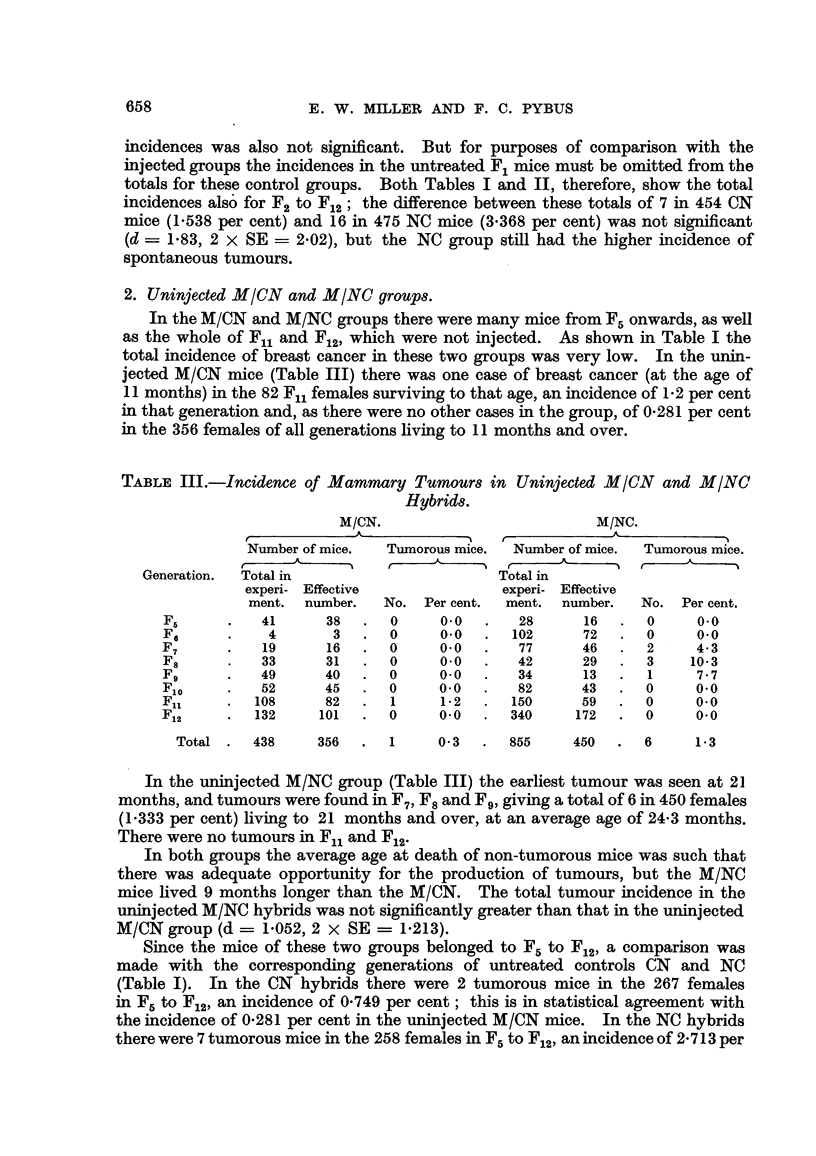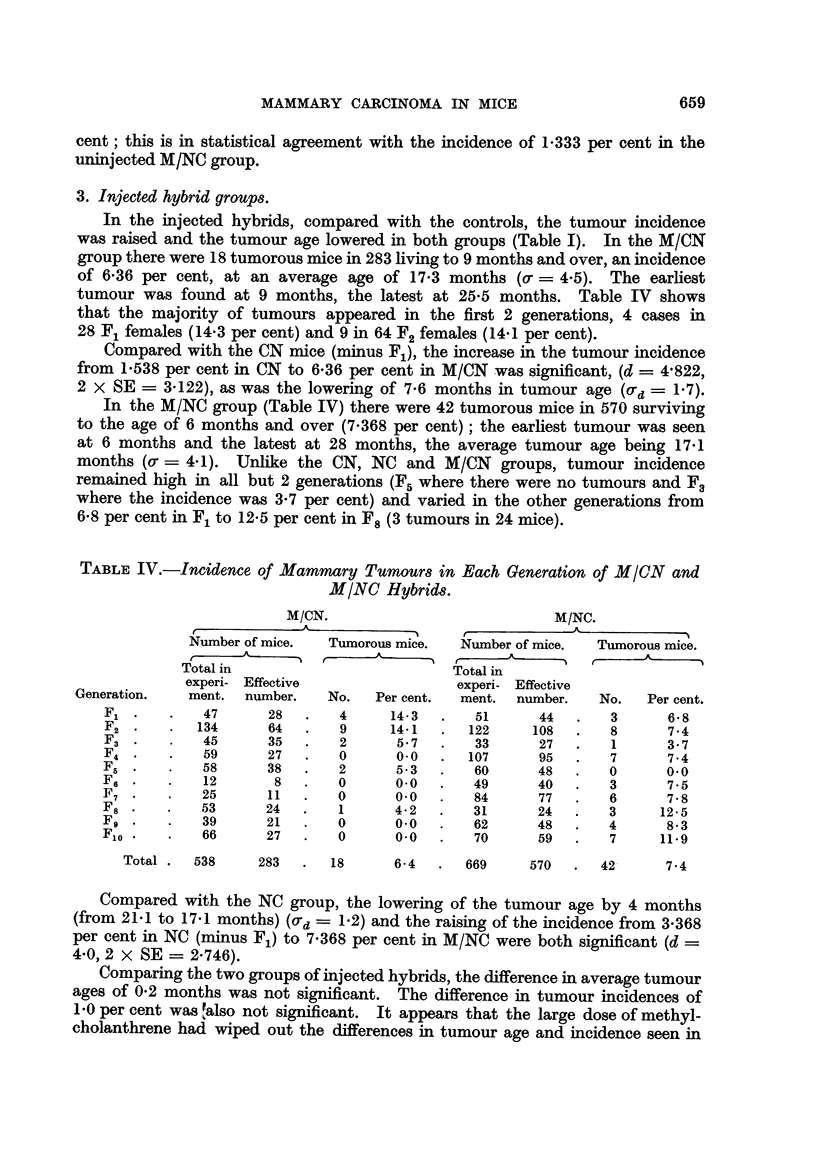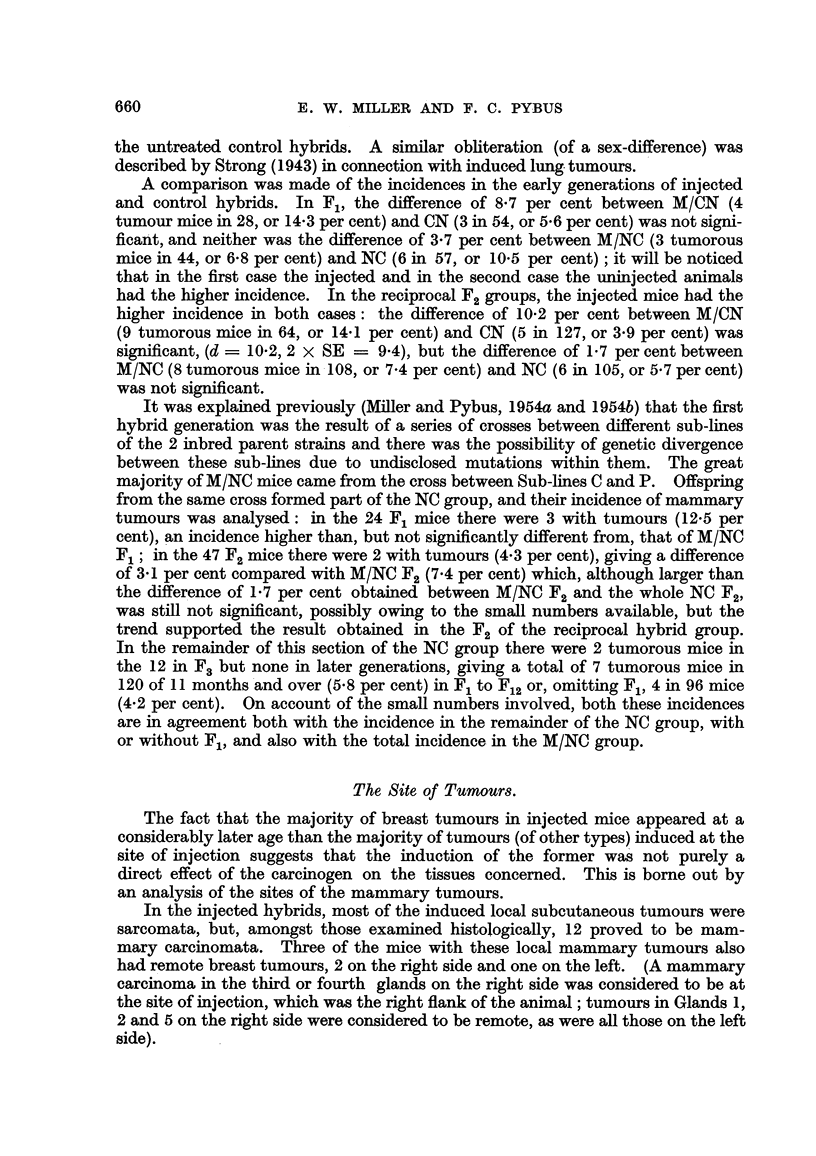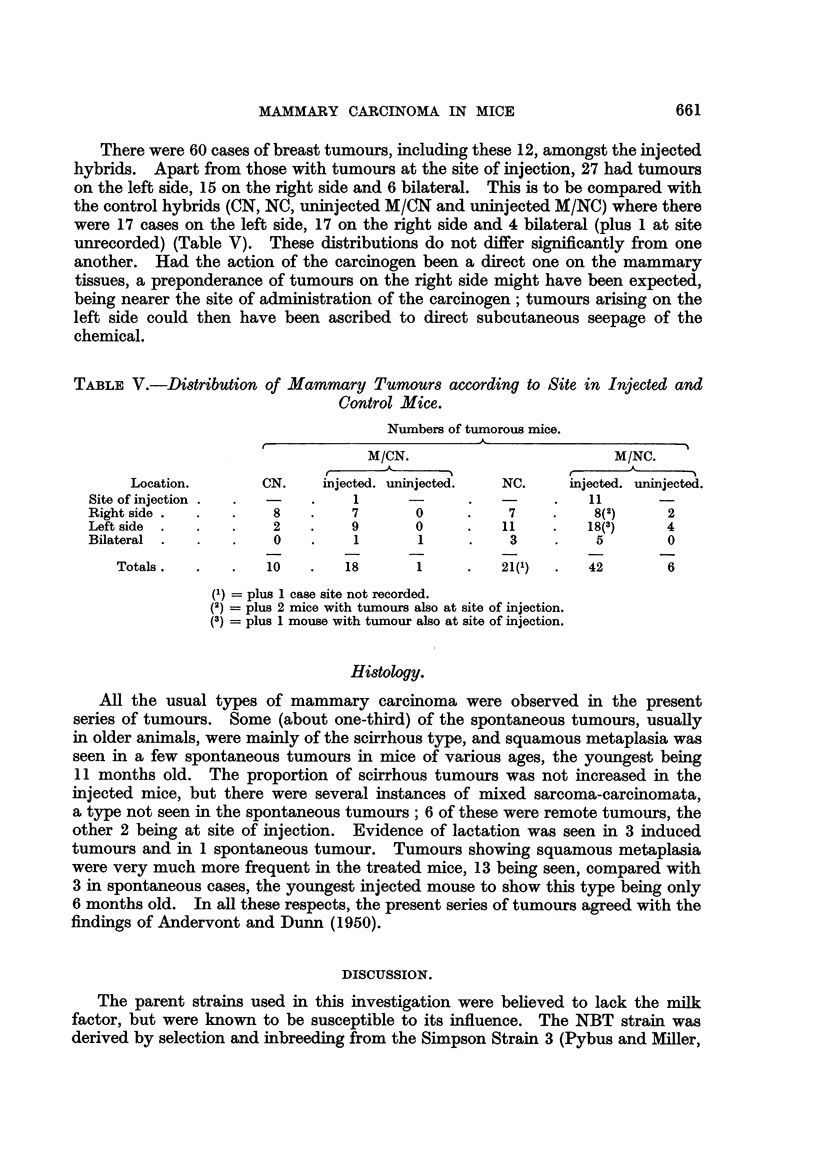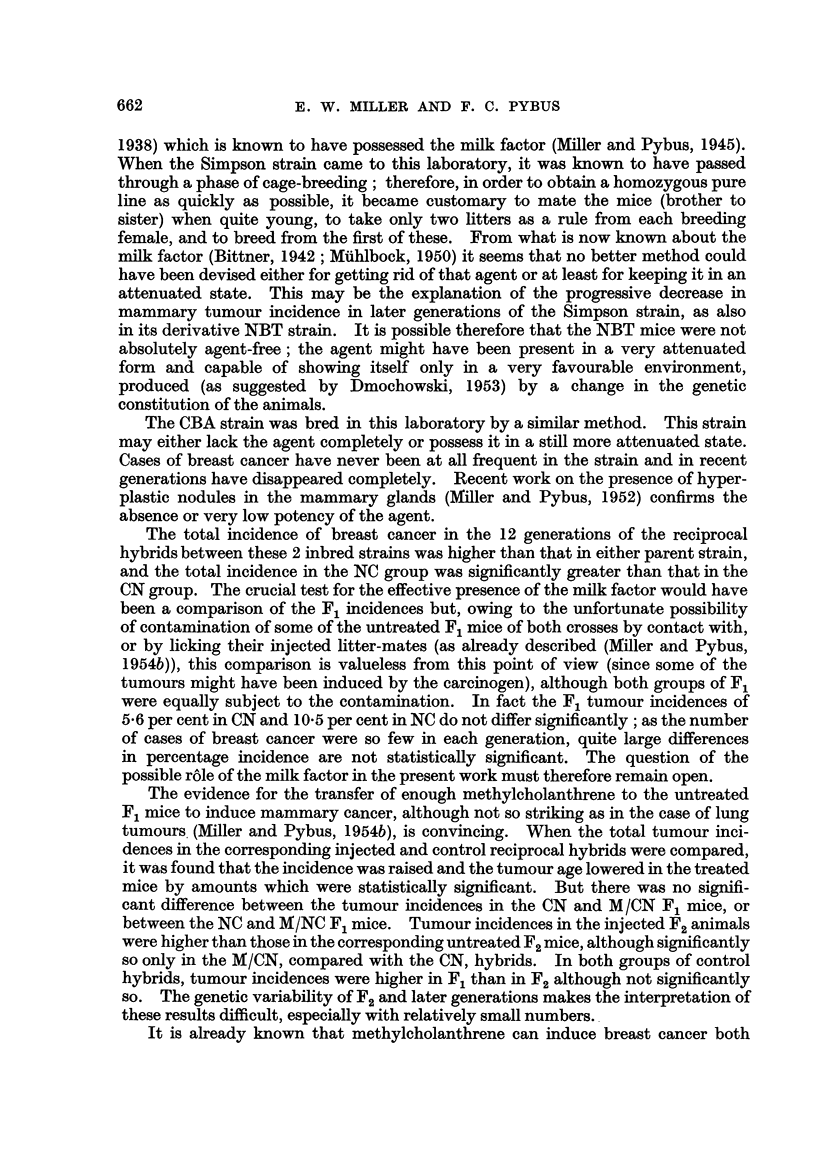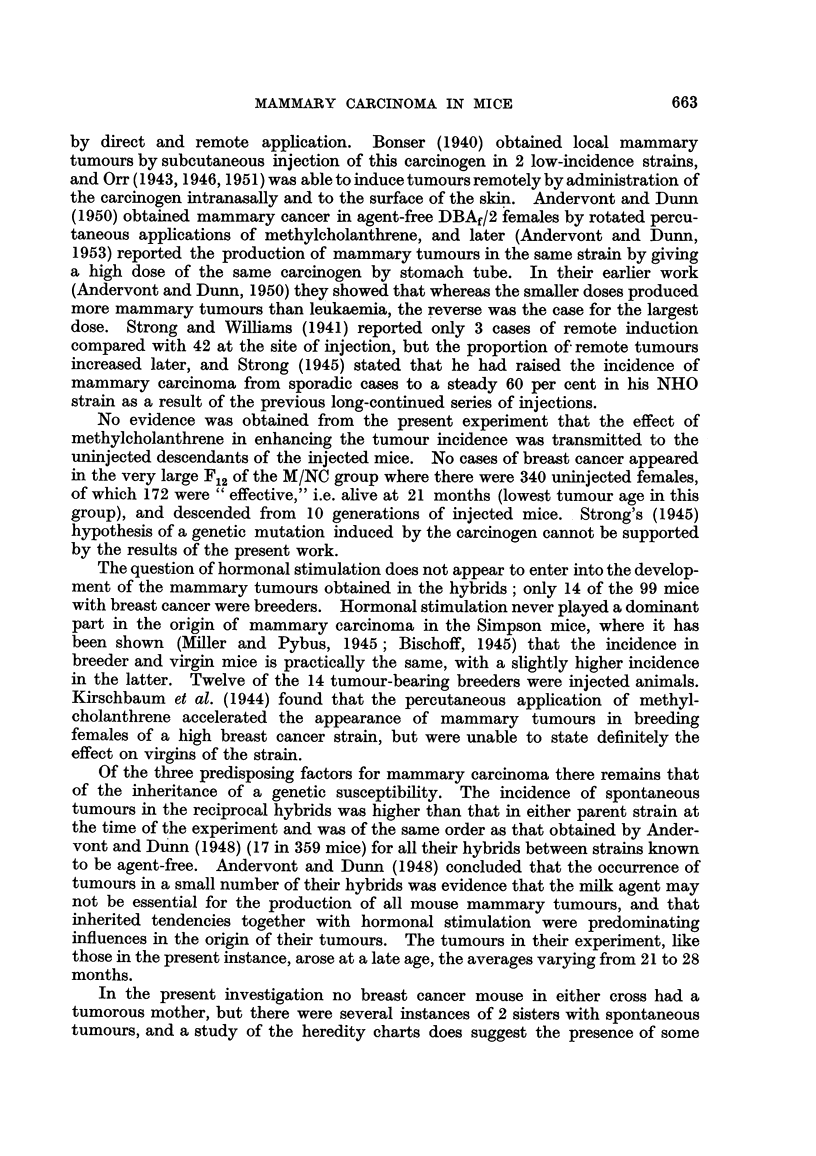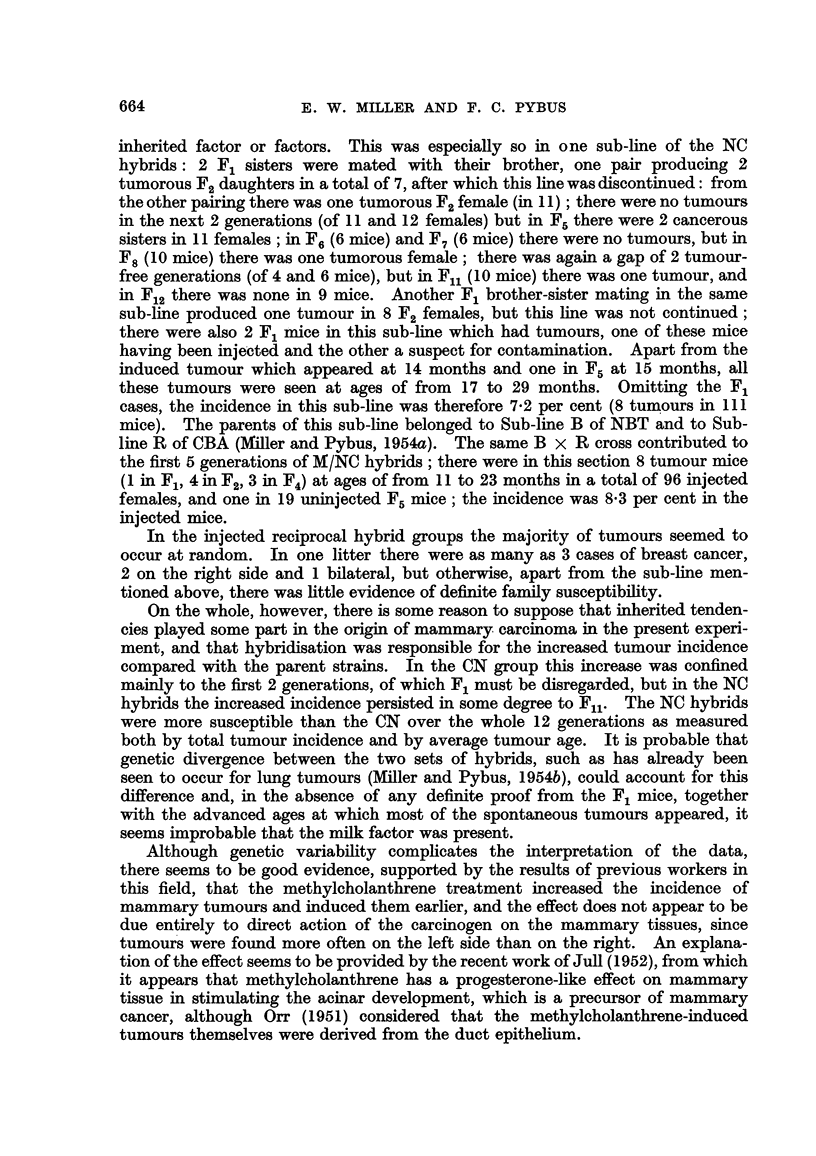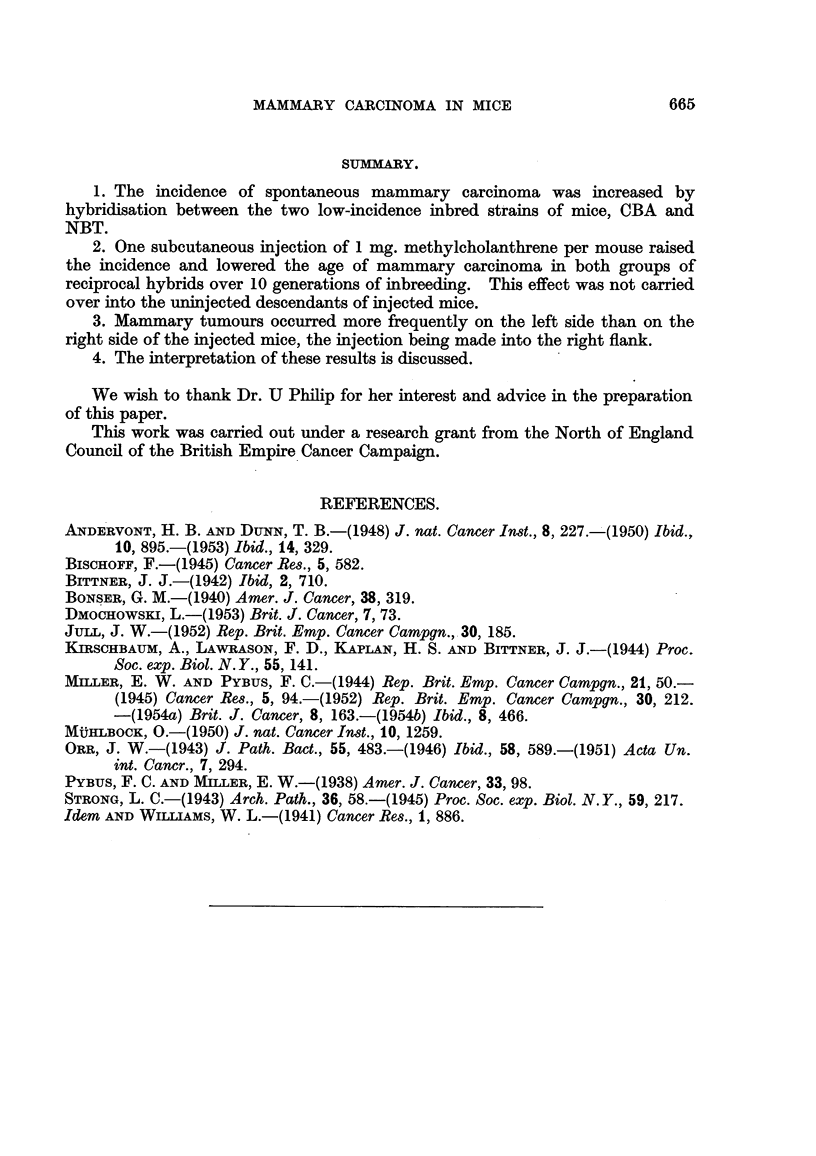# The Effect of Methylcholanthrene on the Incidence of Mammary Carcinoma in Two Inbred Strains of Mice and their Reciprocal Hybrids

**DOI:** 10.1038/bjc.1954.71

**Published:** 1954-12

**Authors:** E. W. Miller, F. C. Pybus


### THE EFFECT OF METHYLCHOLANTHRENE ON THE INCIDENCE OF MAMMARY CARCINOMA IN TWO INBRED STRAINS OF MICE AND THEIR RECIPROCAL HYBRIDS.

E. W. MILLER AND F. C. PYBUS.

*From The J. H. Burn Research Laboratory, Royal Victoria Infirmary, Newcastle upon Tyne.*

Received for publication September 13, 1954.

THIS is the third of a series of reports on an experiment in which 1 mg. methylcholanthrene in 0·1 c.c. sesame oil was injected subcutaneously into the right flank of a number of two-months-old mice, belonging to the inbred strains CBA and NBT and to 10 inbred generations of their reciprocal hybrids beginning with $F_1$. A further 2 generations of untreated hybrids were raised, and the controls were the corresponding 12 generations of untreated mice of the same descent, originating from the untreated $F_1$ litter-mates of the injected hybrids as already described (Miller and Pybus, 1954*a*). In both groups of injected hybrids there were some litters from $F_5$ onwards which were not injected and not bred from ; these served as further controls. The reciprocal hybrids were named the CBA/NBT or CN, and the NBT/CBA or NC strains ; the prefix M was used to denote the injected groups and the uninjected descendants of injected mice. After injection, the mice of each pure strain were bred from, for one generation only, to give the M/NBT $F_1$ and M/CBA $F_1$ groups, this generation remaining untreated. During the period of the experiment there were available as pure strain controls 254 females and 295 males of the CBA strain, and 309 females and 363 males of the NBT strain.

At one time the incidence of sporadic mammary tumours in the CBA strain was about 5 per cent, but latterly had declined. The NBT mice, although derived from the high mammary tumour Simpson strain, had never had a high incidence of breast cancer and had produced no case since the seventeenth generation ; NBT mice from Generations 28 and 29 were used as parents of the hybrids, and mice from Generations 28 to 30 were injected, in the present experiment. During the period of the experiment no mammary tumours appeared in the untreated mice of either inbred strain. Both inbred strains were believed to lack the milk factor, but had been proved to be susceptible to its influence (Miller and Pybus, 1944, 1945). The origin of the mice used in the present work was given in detail in a previous communication (Miller and Pybus, 1954*a*).

Throughout this paper, the word "tumour" without qualification denotes " mammary tumour " and the incidences are based in all cases on the effective numbers of mice, i.e. the numbers alive in each group at the time of discovery of the earliest mammary tumour in that group. As will be seen from the various tables, many mice in all groups and especially in the injected groups died before reaching tumour age. " Tumour age " is subject to the qualification mentioned in the previous communications (Miller and Pybus, 1954*a* and 1954*b*) since, although a superficial tumour, the neoplasm could not be identified for certain until the mouse was killed and the tumour sectioned; mice were allowed to live as long as possible in order that remote effects of the carcinogen might be discovered. Owing to the large numbers of mice in the experiment, tumours, especially in later generations, were charted only at death instead of at their first appearance, but the mice of all groups were equally subject to this error, and the "tumour ages" in the different groups are therefore legitimately comparable and are an indication of the relative susceptibilities of the different groups.

Since this paper deals with mammary tumours, and no instance of a male mouse with this type of tumour occurred during the experiment, the numbers of mice given refer only to females.

### RESULTS.

*Pure strains.*—There were no tumours in the treated M/CBA mice or in their untreated daughters. There was one tumour (1·7 per cent) at the age of 5 months in an M/NBT mouse, and one (1·2 per cent) at the age of 17 months in an uninjected daughter (M/NBT $F_1$) (Table I).

*Reciprocal hybrid strains.*—Tumours appeared in all groups of hybrids, the CN and the reciprocal NC, and the treated M/CN and M/NC groups (Table I). Of the total of 99 tumours hybrid mice, only 14 were breeders; in the Simpson (otherwise Marsh Buffalo) strain, virgin and breeding females have similar tumour incidences (Bischoff, 1945; Miller and Pybus, 1945).

### 1. *Control hybrid groups.*

Table I shows the tumour incidences and the ages at death of tumorous and non-tumorous mice in the various groups. In the 12 generations of CN hybrids there were 10 mice with breast tumours (1·965 per cent) in a total of 509 females of 15 months and over (15 months being the earliest age at which a tumour was found in this group), at an average age of 24·9 months ($\sigma = 4{\cdot}4$); the latest tumour occurred at 30 months. As shown in Table II, 8 of these cases appeared in $F_1$ and $F_2$, where the incidences were 5·6 per cent (3 in 54 $F_1$ mice) and 3·9 per cent (5 in 127 $F_2$ mice) respectively, and the remaining 2 in $F_9$ and $F_{11}$.

TABLE I.—*Incidence of Mammary Tumours in Untreated and Methylcholanthrene-injected Female Mice of Two Inbred Strains and their Reciprocal Hybrids.*

| Strain and Generations. | Number of mice. Total in experiment. | Number of mice. Effective number. | Tumorous mice. Number. | Tumorous mice. Per cent. | Tumorous mice. Age at death (months). Average. | Tumorous mice. Age at death (months). Range. | Non-tumorous mice. Age at death (months). Average. | Non-tumorous mice. Age at death (months). Range. |
|---|---|---|---|---|---|---|---|---|
| CBA controls | 254 | — | 0 | 0·0 | — | — | — | — |
| M/CBA injected | 51 | — | 0 | 0·0 | — | — | — | — |
| M/CBA $F_1$ uninjected | 168 | — | 0 | 0·0 | — | — | — | — |
| NBT controls | 309 | — | 0 | 0·0 | — | — | — | — |
| M/NBT injected | 59 | 59 | 1 | 1·7 | 5·5 | 5·5 | — | — |
| M/NBT $F_1$ uninjected | 81 | 36 | 1 | 2·8 | 17·0 | 17·0 | — | — |
| CN controls $F_1$–$F_{12}$ | 697 | 509 | 10 | 2·0 | 24·9 | 15·0–30·0 | 22·9 | 15·0–34·0 |
| $F_2$–$F_{12}$ | 639 | 455 | 7 | 1·5 | 22·7 | 15·0–26·0 | — | — |
| $F_5$–$F_{12}$ | 418 | 267 | 2 | 0·7 | 20·5 | 15·0–26·0 | — | — |
| M/CN uninjected $F_5$–$F_{12}$ | 438 | 356 | 1 | 0·3 | 11·0 | 11·0 | 14·9 | 11·0–25·0 |
| M/CN injected $F_1$–$F_{10}$ | 538 | 283 | 18 | 6·4 | 17·3 | 9·0–25·5 | 14·8 | 9·0–28·0 |
| NC controls $F_1$–$F_{12}$ | 575 | 532 | 22 | 4·1 | 21·1 | 11·0–29·5 | 21·8 | 11·0–36·0 |
| $F_2$–$F_{12}$ | 517 | 475 | 16 | 3·4 | 19·3 | 11·0–26·0 | — | — |
| $F_5$–$F_{12}$ | 290 | 258 | 7 | 2·7 | 18·9 | 13·0–24·0 | — | — |
| M/NC uninjected $F_5$–$F_{12}$ | 855 | 450 | 6 | 1·3 | 24·3 | 21·0–27·0 | 23·6 | 21·0–29·0 |
| M/NC injected $F_1$–$F_{10}$ | 669 | 570 | 42 | 7·4 | 17·1 | 6·0–28·0 | 10·8 | 6·0–31·0 |

In the 12 generations of NC hybrids (Table I) there were 22 cases of breast cancer (4·135 per cent) in a total of 532 females of 11 months and over, at an average age of 21·1 months ($\sigma = 4{\cdot}8$). The earliest tumour in this group was found at 11 months and the latest at 29·5 months. Table II shows that again the majority of cases appeared in $F_1$ and $F_2$ (6 in 57 $F_1$ mice or 10·5 per cent, and 6 in 105 $F_2$ mice or 5·7 per cent), the remainder being scattered through the other generations, 3 occurring in $F_5$ (7·9 per cent, in 38 mice).

TABLE II.—*Incidence of Mammary Tumours in Each Generation of CN and NC Hybrids.*

| Generation. | CN. Number of mice. Total in experiment. | CN. Number of mice. Effective number. | CN. Tumorous mice. No. | CN. Tumorous mice. Per cent. | NC. Number of mice. Total in experiment. | NC. Number of mice. Effective number. | NC. Tumorous mice. No. | NC. Tumorous mice. Per cent. |
|---|---|---|---|---|---|---|---|---|
| $F_1$ | 58 | 54 | 3 | 5·6 | 58 | 57 | 6 | 10·5 |
| $F_2$ | 145 | 127 | 5 | 3·9 | 109 | 105 | 6 | 5·7 |
| $F_3$ | 41 | 33 | 0 | 0·0 | 55 | 54 | 2 | 3·7 |
| $F_4$ | 37 | 28 | 0 | 0·0 | 63 | 58 | 1 | 1·7 |
| $F_5$ | 33 | 23 | 0 | 0·0 | 42 | 38 | 3 | 7·9 |
| $F_6$ | 29 | 21 | 0 | 0·0 | 48 | 43 | 1 | 2·3 |
| $F_7$ | 24 | 16 | 0 | 0·0 | 24 | 22 | 0 | 0·0 |
| $F_8$ | 38 | 31 | 0 | 0·0 | 50 | 47 | 1 | 2·1 |
| $F_9$ | 42 | 33 | 1 | 3·0 | 39 | 33 | 1 | 3·0 |
| $F_{10}$ | 59 | 37 | 0 | 0·0 | 33 | 27 | 0 | 0·0 |
| $F_{11}$ | 66 | 30 | 1 | 3·3 | 26 | 21 | 1 | 4·8 |
| $F_{12}$ | 127 | 76 | 0 | 0·0 | 28 | 27 | 0 | 0·0 |
| Totals : | | | | | | | | |
| $F_1$–$F_{12}$ | 697 | 509 | 10 | 2·0 | 575 | 532 | 22 | 4·1 |
| $F_2$–$F_{12}$ | 639 | 455 | 7 | 1·5 | 517 | 475 | 16 | 3·4 |
| $F_5$–$F_{12}$ | 418 | 267 | 2 | 0·7 | 290 | 258 | 7 | 2·7 |

The differences of 2·17 per cent in the tumour incidences (twice the standard error of the difference = 2·12) and of 3·8 months in the average tumour ages ($\sigma_d = 1{\cdot}7$) in the reciprocal hybrids were both statistically significant. The total NC group therefore had a mammary tumour incidence more than twice that of the total CN group, and tumours appeared nearly 4 months earlier in the NC mice. In both groups, as shown in Table I, the non-tumorous mice lived to about the same age as the tumorous mice.

In both groups the tumour incidences were higher in $F_1$ than in $F_2$, but not significantly so. In the preceding paper of this series (Miller and Pybus, 1954*b*) it was pointed out how a possible contamination of some uninjected $F_1$ mice could have taken place by contact with, or by licking, their injected litter-mates. There was no means of telling which mice might have been contaminated, nor whether the amount of carcinogen transferred was always sufficient to induce neoplasms ; in the case of lung tumours there was good reason to believe that the quantity was enough, but in the case of forestomach papillomata it seemed to be inadequate. In the present instance, since both groups were equally subject to such possible contamination in $F_1$, a comparison between the incidences in the reciprocal $F_1$ mice would still be valid : in fact, owing to the small numbers, this difference was not significant, and the difference between the reciprocal $F_2$ tumour incidences was also not significant. But for purposes of comparison with the injected groups the incidences in the untreated $F_1$ mice must be omitted from the totals for these control groups. Both Tables I and II, therefore, show the total incidences also for $F_2$ to $F_{12}$; the difference between these totals of 7 in 454 CN mice (1·538 per cent) and 16 in 475 NC mice (3·368 per cent) was not significant ($d$ = 1·83, 2 × SE = 2·02), but the NC group still had the higher incidence of spontaneous tumours.

2. *Uninjected M/CN and M/NC groups.*

In the M/CN and M/NC groups there were many mice from $F_5$ onwards, as well as the whole of $F_{11}$ and $F_{12}$, which were not injected. As shown in Table I the total incidence of breast cancer in these two groups was very low. In the uninjected M/CN mice (Table III) there was one case of breast cancer (at the age of 11 months) in the 82 $F_{11}$ females surviving to that age, an incidence of 1·2 per cent in that generation and, as there were no other cases in the group, of 0·281 per cent in the 356 females of all generations living to 11 months and over.

TABLE III.—*Incidence of Mammary Tumours in Uninjected M/CN and M/NC Hybrids.*

| | M/CN. | | | | M/NC. | | | |
|---|---|---|---|---|---|---|---|---|
| | Number of mice. | | Tumorous mice. | | Number of mice. | | Tumorous mice. | |
| Generation. | Total in experiment. | Effective number. | No. | Per cent. | Total in experiment. | Effective number. | No. | Per cent. |
| $F_5$ | 41 | 38 | 0 | 0·0 | 28 | 16 | 0 | 0·0 |
| $F_6$ | 4 | 3 | 0 | 0·0 | 102 | 72 | 0 | 0·0 |
| $F_7$ | 19 | 16 | 0 | 0·0 | 77 | 46 | 2 | 4·3 |
| $F_8$ | 33 | 31 | 0 | 0·0 | 42 | 29 | 3 | 10·3 |
| $F_9$ | 49 | 40 | 0 | 0·0 | 34 | 13 | 1 | 7·7 |
| $F_{10}$ | 52 | 45 | 0 | 0·0 | 82 | 43 | 0 | 0·0 |
| $F_{11}$ | 108 | 82 | 1 | 1·2 | 150 | 59 | 0 | 0·0 |
| $F_{12}$ | 132 | 101 | 0 | 0·0 | 340 | 172 | 0 | 0·0 |
| Total | 438 | 356 | 1 | 0·3 | 855 | 450 | 6 | 1·3 |

In the uninjected M/NC group (Table III) the earliest tumour was seen at 21 months, and tumours were found in $F_7$, $F_8$ and $F_9$, giving a total of 6 in 450 females (1·333 per cent) living to 21 months and over, at an average age of 24·3 months. There were no tumours in $F_{11}$ and $F_{12}$.

In both groups the average age at death of non-tumorous mice was such that there was adequate opportunity for the production of tumours, but the M/NC mice lived 9 months longer than the M/CN. The total tumour incidence in the uninjected M/NC hybrids was not significantly greater than that in the uninjected M/CN group (d = 1·052, 2 × SE = 1·213).

Since the mice of these two groups belonged to $F_5$ to $F_{12}$, a comparison was made with the corresponding generations of untreated controls CN and NC (Table I). In the CN hybrids there were 2 tumorous mice in the 267 females in $F_5$ to $F_{12}$, an incidence of 0·749 per cent; this is in statistical agreement with the incidence of 0·281 per cent in the uninjected M/CN mice. In the NC hybrids there were 7 tumorous mice in the 258 females in $F_5$ to $F_{12}$, an incidence of 2·713 per cent ; this is in statistical agreement with the incidence of 1·333 per cent in the uninjected M/NC group.

3. *Injected hybrid groups.*

In the injected hybrids, compared with the controls, the tumour incidence was raised and the tumour age lowered in both groups (Table I). In the M/CN group there were 18 tumorous mice in 283 living to 9 months and over, an incidence of 6·36 per cent, at an average age of 17·3 months ($\sigma = 4{\cdot}5$). The earliest tumour was found at 9 months, the latest at 25·5 months. Table IV shows that the majority of tumours appeared in the first 2 generations, 4 cases in 28 $F_1$ females (14·3 per cent) and 9 in 64 $F_2$ females (14·1 per cent).

Compared with the CN mice (minus $F_1$), the increase in the tumour incidence from 1·538 per cent in CN to 6·36 per cent in M/CN was significant, ($d = 4{\cdot}822$, $2 \times \text{SE} = 3{\cdot}122$), as was the lowering of 7·6 months in tumour age ($\sigma_d = 1{\cdot}7$).

In the M/NC group (Table IV) there were 42 tumorous mice in 570 surviving to the age of 6 months and over (7·368 per cent) ; the earliest tumour was seen at 6 months and the latest at 28 months, the average tumour age being 17·1 months ($\sigma = 4{\cdot}1$). Unlike the CN, NC and M/CN groups, tumour incidence remained high in all but 2 generations ($F_5$ where there were no tumours and $F_3$ where the incidence was 3·7 per cent) and varied in the other generations from 6·8 per cent in $F_1$ to 12·5 per cent in $F_8$ (3 tumours in 24 mice).

TABLE IV.—*Incidence of Mammary Tumours in Each Generation of M/CN and M/NC Hybrids.*

| | M/CN. | | | | M/NC. | | | |
|---|---|---|---|---|---|---|---|---|
| | Number of mice. | | Tumorous mice. | | Number of mice. | | Tumorous mice. | |
| Generation. | Total in experiment. | Effective number. | No. | Per cent. | Total in experiment. | Effective number. | No. | Per cent. |
| $F_1$ | 47 | 28 | 4 | 14·3 | 51 | 44 | 3 | 6·8 |
| $F_2$ | 134 | 64 | 9 | 14·1 | 122 | 108 | 8 | 7·4 |
| $F_3$ | 45 | 35 | 2 | 5·7 | 33 | 27 | 1 | 3·7 |
| $F_4$ | 59 | 27 | 0 | 0·0 | 107 | 95 | 7 | 7·4 |
| $F_5$ | 58 | 38 | 2 | 5·3 | 60 | 48 | 0 | 0·0 |
| $F_6$ | 12 | 8 | 0 | 0·0 | 49 | 40 | 3 | 7·5 |
| $F_7$ | 25 | 11 | 0 | 0·0 | 84 | 77 | 6 | 7·8 |
| $F_8$ | 53 | 24 | 1 | 4·2 | 31 | 24 | 3 | 12·5 |
| $F_9$ | 39 | 21 | 0 | 0·0 | 62 | 48 | 4 | 8·3 |
| $F_{10}$ | 66 | 27 | 0 | 0·0 | 70 | 59 | 7 | 11·9 |
| Total | 538 | 283 | 18 | 6·4 | 669 | 570 | 42 | 7·4 |

Compared with the NC group, the lowering of the tumour age by 4 months (from 21·1 to 17·1 months) ($\sigma_d = 1{\cdot}2$) and the raising of the incidence from 3·368 per cent in NC (minus $F_1$) to 7·368 per cent in M/NC were both significant ($d = 4{\cdot}0$, $2 \times \text{SE} = 2{\cdot}746$).

Comparing the two groups of injected hybrids, the difference in average tumour ages of 0·2 months was not significant. The difference in tumour incidences of 1·0 per cent was also not significant. It appears that the large dose of methylcholanthrene had wiped out the differences in tumour age and incidence seen in the untreated control hybrids. A similar obliteration (of a sex-difference) was described by Strong (1943) in connection with induced lung tumours.

A comparison was made of the incidences in the early generations of injected and control hybrids. In $F_1$, the difference of 8·7 per cent between M/CN (4 tumour mice in 28, or 14·3 per cent) and CN (3 in 54, or 5·6 per cent) was not significant, and neither was the difference of 3·7 per cent between M/NC (3 tumorous mice in 44, or 6·8 per cent) and NC (6 in 57, or 10·5 per cent); it will be noticed that in the first case the injected and in the second case the uninjected animals had the higher incidence. In the reciprocal $F_2$ groups, the injected mice had the higher incidence in both cases: the difference of 10·2 per cent between M/CN (9 tumorous mice in 64, or 14·1 per cent) and CN (5 in 127, or 3·9 per cent) was significant, ($d = 10{\cdot}2$, $2 \times SE = 9{\cdot}4$), but the difference of 1·7 per cent between M/NC (8 tumorous mice in 108, or 7·4 per cent) and NC (6 in 105, or 5·7 per cent) was not significant.

It was explained previously (Miller and Pybus, 1954*a* and 1954*b*) that the first hybrid generation was the result of a series of crosses between different sub-lines of the 2 inbred parent strains and there was the possibility of genetic divergence between these sub-lines due to undisclosed mutations within them. The great majority of M/NC mice came from the cross between Sub-lines C and P. Offspring from the same cross formed part of the NC group, and their incidence of mammary tumours was analysed: in the 24 $F_1$ mice there were 3 with tumours (12·5 per cent), an incidence higher than, but not significantly different from, that of M/NC $F_1$; in the 47 $F_2$ mice there were 2 with tumours (4·3 per cent), giving a difference of 3·1 per cent compared with M/NC $F_2$ (7·4 per cent) which, although larger than the difference of 1·7 per cent obtained between M/NC $F_2$ and the whole NC $F_2$, was still not significant, possibly owing to the small numbers available, but the trend supported the result obtained in the $F_2$ of the reciprocal hybrid group. In the remainder of this section of the NC group there were 2 tumorous mice in the 12 in $F_3$ but none in later generations, giving a total of 7 tumorous mice in 120 of 11 months and over (5·8 per cent) in $F_1$ to $F_{12}$ or, omitting $F_1$, 4 in 96 mice (4·2 per cent). On account of the small numbers involved, both these incidences are in agreement both with the incidence in the remainder of the NC group, with or without $F_1$, and also with the total incidence in the M/NC group.

### *The Site of Tumours.*

The fact that the majority of breast tumours in injected mice appeared at a considerably later age than the majority of tumours (of other types) induced at the site of injection suggests that the induction of the former was not purely a direct effect of the carcinogen on the tissues concerned. This is borne out by an analysis of the sites of the mammary tumours.

In the injected hybrids, most of the induced local subcutaneous tumours were sarcomata, but, amongst those examined histologically, 12 proved to be mammary carcinomata. Three of the mice with these local mammary tumours also had remote breast tumours, 2 on the right side and one on the left. (A mammary carcinoma in the third or fourth glands on the right side was considered to be at the site of injection, which was the right flank of the animal; tumours in Glands 1, 2 and 5 on the right side were considered to be remote, as were all those on the left side).

There were 60 cases of breast tumours, including these 12, amongst the injected hybrids. Apart from those with tumours at the site of injection, 27 had tumours on the left side, 15 on the right side and 6 bilateral. This is to be compared with the control hybrids (CN, NC, uninjected M/CN and uninjected M/NC) where there were 17 cases on the left side, 17 on the right side and 4 bilateral (plus 1 at site unrecorded) (Table V). These distributions do not differ significantly from one another. Had the action of the carcinogen been a direct one on the mammary tissues, a preponderance of tumours on the right side might have been expected, being nearer the site of administration of the carcinogen ; tumours arising on the left side could then have been ascribed to direct subcutaneous seepage of the chemical.

TABLE V.—*Distribution of Mammary Tumours according to Site in Injected and Control Mice.*

| | Numbers of tumorous mice. | | | | | |
|---|---|---|---|---|---|---|
| | | M/CN. | | | M/NC. | |
| Location. | CN. | injected. | uninjected. | NC. | injected. | uninjected. |
| Site of injection | — | 1 | — | — | 11 | — |
| Right side | 8 | 7 | 0 | 7 | 8(²) | 2 |
| Left side | 2 | 9 | 0 | 11 | 18(³) | 4 |
| Bilateral | 0 | 1 | 1 | 3 | 5 | 0 |
| Totals | 10 | 18 | 1 | 21(¹) | 42 | 6 |

(¹) = plus 1 case site not recorded.
(²) = plus 2 mice with tumours also at site of injection.
(³) = plus 1 mouse with tumour also at site of injection.

### *Histology.*

All the usual types of mammary carcinoma were observed in the present series of tumours. Some (about one-third) of the spontaneous tumours, usually in older animals, were mainly of the scirrhous type, and squamous metaplasia was seen in a few spontaneous tumours in mice of various ages, the youngest being 11 months old. The proportion of scirrhous tumours was not increased in the injected mice, but there were several instances of mixed sarcoma-carcinomata, a type not seen in the spontaneous tumours ; 6 of these were remote tumours, the other 2 being at site of injection. Evidence of lactation was seen in 3 induced tumours and in 1 spontaneous tumour. Tumours showing squamous metaplasia were very much more frequent in the treated mice, 13 being seen, compared with 3 in spontaneous cases, the youngest injected mouse to show this type being only 6 months old. In all these respects, the present series of tumours agreed with the findings of Andervont and Dunn (1950).

### DISCUSSION.

The parent strains used in this investigation were believed to lack the milk factor, but were known to be susceptible to its influence. The NBT strain was derived by selection and inbreeding from the Simpson Strain 3 (Pybus and Miller,

1938) which is known to have possessed the milk factor (Miller and Pybus, 1945). When the Simpson strain came to this laboratory, it was known to have passed through a phase of cage-breeding ; therefore, in order to obtain a homozygous pure line as quickly as possible, it became customary to mate the mice (brother to sister) when quite young, to take only two litters as a rule from each breeding female, and to breed from the first of these. From what is now known about the milk factor (Bittner, 1942 ; Mühlbock, 1950) it seems that no better method could have been devised either for getting rid of that agent or at least for keeping it in an attenuated state. This may be the explanation of the progressive decrease in mammary tumour incidence in later generations of the Simpson strain, as also in its derivative NBT strain. It is possible therefore that the NBT mice were not absolutely agent-free ; the agent might have been present in a very attenuated form and capable of showing itself only in a very favourable environment, produced (as suggested by Dmochowski, 1953) by a change in the genetic constitution of the animals.

The CBA strain was bred in this laboratory by a similar method. This strain may either lack the agent completely or possess it in a still more attenuated state. Cases of breast cancer have never been at all frequent in the strain and in recent generations have disappeared completely. Recent work on the presence of hyperplastic nodules in the mammary glands (Miller and Pybus, 1952) confirms the absence or very low potency of the agent.

The total incidence of breast cancer in the 12 generations of the reciprocal hybrids between these 2 inbred strains was higher than that in either parent strain, and the total incidence in the NC group was significantly greater than that in the CN group. The crucial test for the effective presence of the milk factor would have been a comparison of the $F_1$ incidences but, owing to the unfortunate possibility of contamination of some of the untreated $F_1$ mice of both crosses by contact with, or by licking their injected litter-mates (as already described (Miller and Pybus, 1954*b*)), this comparison is valueless from this point of view (since some of the tumours might have been induced by the carcinogen), although both groups of $F_1$ were equally subject to the contamination. In fact the $F_1$ tumour incidences of 5·6 per cent in CN and 10·5 per cent in NC do not differ significantly ; as the number of cases of breast cancer were so few in each generation, quite large differences in percentage incidence are not statistically significant. The question of the possible rôle of the milk factor in the present work must therefore remain open.

The evidence for the transfer of enough methylcholanthrene to the untreated $F_1$ mice to induce mammary cancer, although not so striking as in the case of lung tumours (Miller and Pybus, 1954*b*), is convincing. When the total tumour incidences in the corresponding injected and control reciprocal hybrids were compared, it was found that the incidence was raised and the tumour age lowered in the treated mice by amounts which were statistically significant. But there was no significant difference between the tumour incidences in the CN and M/CN $F_1$ mice, or between the NC and M/NC $F_1$ mice. Tumour incidences in the injected $F_2$ animals were higher than those in the corresponding untreated $F_2$ mice, although significantly so only in the M/CN, compared with the CN, hybrids. In both groups of control hybrids, tumour incidences were higher in $F_1$ than in $F_2$ although not significantly so. The genetic variability of $F_2$ and later generations makes the interpretation of these results difficult, especially with relatively small numbers.

It is already known that methylcholanthrene can induce breast cancer both by direct and remote application. Bonser (1940) obtained local mammary tumours by subcutaneous injection of this carcinogen in 2 low-incidence strains, and Orr (1943, 1946, 1951) was able to induce tumours remotely by administration of the carcinogen intranasally and to the surface of the skin. Andervont and Dunn (1950) obtained mammary cancer in agent-free $DBA_f/2$ females by rotated percutaneous applications of methylcholanthrene, and later (Andervont and Dunn, 1953) reported the production of mammary tumours in the same strain by giving a high dose of the same carcinogen by stomach tube. In their earlier work (Andervont and Dunn, 1950) they showed that whereas the smaller doses produced more mammary tumours than leukaemia, the reverse was the case for the largest dose. Strong and Williams (1941) reported only 3 cases of remote induction compared with 42 at the site of injection, but the proportion of remote tumours increased later, and Strong (1945) stated that he had raised the incidence of mammary carcinoma from sporadic cases to a steady 60 per cent in his NHO strain as a result of the previous long-continued series of injections.

No evidence was obtained from the present experiment that the effect of methylcholanthrene in enhancing the tumour incidence was transmitted to the uninjected descendants of the injected mice. No cases of breast cancer appeared in the very large $F_{12}$ of the M/NC group where there were 340 uninjected females, of which 172 were "effective," i.e. alive at 21 months (lowest tumour age in this group), and descended from 10 generations of injected mice. Strong's (1945) hypothesis of a genetic mutation induced by the carcinogen cannot be supported by the results of the present work.

The question of hormonal stimulation does not appear to enter into the development of the mammary tumours obtained in the hybrids ; only 14 of the 99 mice with breast cancer were breeders. Hormonal stimulation never played a dominant part in the origin of mammary carcinoma in the Simpson mice, where it has been shown (Miller and Pybus, 1945 ; Bischoff, 1945) that the incidence in breeder and virgin mice is practically the same, with a slightly higher incidence in the latter. Twelve of the 14 tumour-bearing breeders were injected animals. Kirschbaum *et al.* (1944) found that the percutaneous application of methylcholanthrene accelerated the appearance of mammary tumours in breeding females of a high breast cancer strain, but were unable to state definitely the effect on virgins of the strain.

Of the three predisposing factors for mammary carcinoma there remains that of the inheritance of a genetic susceptibility. The incidence of spontaneous tumours in the reciprocal hybrids was higher than that in either parent strain at the time of the experiment and was of the same order as that obtained by Andervont and Dunn (1948) (17 in 359 mice) for all their hybrids between strains known to be agent-free. Andervont and Dunn (1948) concluded that the occurrence of tumours in a small number of their hybrids was evidence that the milk agent may not be essential for the production of all mouse mammary tumours, and that inherited tendencies together with hormonal stimulation were predominating influences in the origin of their tumours. The tumours in their experiment, like those in the present instance, arose at a late age, the averages varying from 21 to 28 months.

In the present investigation no breast cancer mouse in either cross had a tumorous mother, but there were several instances of 2 sisters with spontaneous tumours, and a study of the heredity charts does suggest the presence of some inherited factor or factors. This was especially so in one sub-line of the NC hybrids: 2 $F_1$ sisters were mated with their brother, one pair producing 2 tumorous $F_2$ daughters in a total of 7, after which this line was discontinued: from the other pairing there was one tumorous $F_2$ female (in 11); there were no tumours in the next 2 generations (of 11 and 12 females) but in $F_5$ there were 2 cancerous sisters in 11 females; in $F_6$ (6 mice) and $F_7$ (6 mice) there were no tumours, but in $F_8$ (10 mice) there was one tumorous female; there was again a gap of 2 tumour-free generations (of 4 and 6 mice), but in $F_{11}$ (10 mice) there was one tumour, and in $F_{12}$ there was none in 9 mice. Another $F_1$ brother-sister mating in the same sub-line produced one tumour in 8 $F_2$ females, but this line was not continued; there were also 2 $F_1$ mice in this sub-line which had tumours, one of these mice having been injected and the other a suspect for contamination. Apart from the induced tumour which appeared at 14 months and one in $F_5$ at 15 months, all these tumours were seen at ages of from 17 to 29 months. Omitting the $F_1$ cases, the incidence in this sub-line was therefore 7·2 per cent (8 tumours in 111 mice). The parents of this sub-line belonged to Sub-line B of NBT and to Sub-line R of CBA (Miller and Pybus, 1954*a*). The same B $\times$ R cross contributed to the first 5 generations of M/NC hybrids; there were in this section 8 tumour mice (1 in $F_1$, 4 in $F_2$, 3 in $F_4$) at ages of from 11 to 23 months in a total of 96 injected females, and one in 19 uninjected $F_5$ mice; the incidence was 8·3 per cent in the injected mice.

In the injected reciprocal hybrid groups the majority of tumours seemed to occur at random. In one litter there were as many as 3 cases of breast cancer, 2 on the right side and 1 bilateral, but otherwise, apart from the sub-line mentioned above, there was little evidence of definite family susceptibility.

On the whole, however, there is some reason to suppose that inherited tendencies played some part in the origin of mammary carcinoma in the present experiment, and that hybridisation was responsible for the increased tumour incidence compared with the parent strains. In the CN group this increase was confined mainly to the first 2 generations, of which $F_1$ must be disregarded, but in the NC hybrids the increased incidence persisted in some degree to $F_{11}$. The NC hybrids were more susceptible than the CN over the whole 12 generations as measured both by total tumour incidence and by average tumour age. It is probable that genetic divergence between the two sets of hybrids, such as has already been seen to occur for lung tumours (Miller and Pybus, 1954*b*), could account for this difference and, in the absence of any definite proof from the $F_1$ mice, together with the advanced ages at which most of the spontaneous tumours appeared, it seems improbable that the milk factor was present.

Although genetic variability complicates the interpretation of the data, there seems to be good evidence, supported by the results of previous workers in this field, that the methylcholanthrene treatment increased the incidence of mammary tumours and induced them earlier, and the effect does not appear to be due entirely to direct action of the carcinogen on the mammary tissues, since tumours were found more often on the left side than on the right. An explanation of the effect seems to be provided by the recent work of Jull (1952), from which it appears that methylcholanthrene has a progesterone-like effect on mammary tissue in stimulating the acinar development, which is a precursor of mammary cancer, although Orr (1951) considered that the methylcholanthrene-induced tumours themselves were derived from the duct epithelium.

SUMMARY.

1. The incidence of spontaneous mammary carcinoma was increased by hybridisation between the two low-incidence inbred strains of mice, CBA and NBT.

2. One subcutaneous injection of 1 mg. methylcholanthrene per mouse raised the incidence and lowered the age of mammary carcinoma in both groups of reciprocal hybrids over 10 generations of inbreeding. This effect was not carried over into the uninjected descendants of injected mice.

3. Mammary tumours occurred more frequently on the left side than on the right side of the injected mice, the injection being made into the right flank.

4. The interpretation of these results is discussed.

We wish to thank Dr. U Philip for her interest and advice in the preparation of this paper.

This work was carried out under a research grant from the North of England Council of the British Empire Cancer Campaign.

REFERENCES.

ANDERVONT, H. B. AND DUNN, T. B.—(1948) *J. nat. Cancer Inst.*, **8**, 227.—(1950) *Ibid.*, **10**, 895.—(1953) *Ibid.*, **14**, 329.

BISCHOFF, F.—(1945) *Cancer Res.*, **5**, 582.

BITTNER, J. J.—(1942) *Ibid*, **2**, 710.

BONSER, G. M.—(1940) *Amer. J. Cancer*, **38**, 319.

DMOCHOWSKI, L.—(1953) *Brit. J. Cancer*, **7**, 73.

JULL, J. W.—(1952) *Rep. Brit. Emp. Cancer Campgn.*, **30**, 185.

KIRSCHBAUM, A., LAWRASON, F. D., KAPLAN, H. S. AND BITTNER, J. J.—(1944) *Proc. Soc. exp. Biol. N.Y.*, **55**, 141.

MILLER, E. W. AND PYBUS, F. C.—(1944) *Rep. Brit. Emp. Cancer Campgn.*, **21**, 50.—(1945) *Cancer Res.*, **5**, 94.—(1952) *Rep. Brit. Emp. Cancer Campgn.*, **30**, 212.—(1954*a*) *Brit. J. Cancer*, **8**, 163.—(1954*b*) *Ibid.*, **8**, 466.

MÜHLBOCK, O.—(1950) *J. nat. Cancer Inst.*, **10**, 1259.

ORR, J. W.—(1943) *J. Path. Bact.*, **55**, 483.—(1946) *Ibid.*, **58**, 589.—(1951) *Acta Un. int. Cancr.*, **7**, 294.

PYBUS, F. C. AND MILLER, E. W.—(1938) *Amer. J. Cancer*, **33**, 98.

STRONG, L. C.—(1943) *Arch. Path.*, **36**, 58.—(1945) *Proc. Soc. exp. Biol. N.Y.*, **59**, 217.

*Idem* AND WILLIAMS, W. L.—(1941) *Cancer Res.*, **1**, 886.